# Data Mining and NIR Spectroscopy in Viticulture: Applications for Plant Phenotyping under Field Conditions †

**DOI:** 10.3390/s16020236

**Published:** 2016-02-16

**Authors:** Salvador Gutiérrez, Javier Tardaguila, Juan Fernández-Novales, Maria P. Diago

**Affiliations:** Instituto de Ciencias de la Vid y del Vino (University of La Rioja, CSIC, Gobierno de La Rioja) Ctra. De Burgos Km, 6, 26007 Logroño, Spain; salvador.gutierrez@unirioja.es (S.G.); javier.tardaguila@unirioja.es (J.T.); juan.fernandezn@unirioja.es (J.F.-N.)

**Keywords:** variety classification, plant water status, non-destructive, SVM, rotation forest, regression tree, stem water potential

## Abstract

Plant phenotyping is a very important topic in agriculture. In this context, data mining strategies may be applied to agricultural data retrieved with new non-invasive devices, with the aim of yielding useful, reliable and objective information. This work presents some applications of machine learning algorithms along with in-field acquired NIR spectral data for plant phenotyping in viticulture, specifically for grapevine variety discrimination and assessment of plant water status. Support vector machine (SVM), rotation forests and M5 trees models were built using NIR spectra acquired in the field directly on the adaxial side of grapevine leaves, with a non-invasive portable spectrophotometer working in the spectral range between 1600 and 2400 nm. The *ν*-SVM algorithm was used for the training of a model for varietal classification. The classifiers’ performance for the 10 varieties reached, for cross- and external validations, the 88.7% and 92.5% marks, respectively. For water stress assessment, the models developed using the absorbance spectra of six varieties yielded the same determination coefficient for both cross- and external validations (*R*^2^ = 0.84; RMSEs of 0.164 and 0.165 MPa, respectively). Furthermore, a variety-specific model trained only with samples of Tempranillo from two different vintages yielded R^2^ = 0.76 and RMSE of 0.16 MPa for cross-validation and R^2^ = 0.79, RMSE of 0.17 MPa for external validation. These results show the power of the combined use of data mining and non-invasive NIR sensing for in-field grapevine phenotyping and their usefulness for the wine industry and precision viticulture implementations.

## 1. Introduction

In the context of the current worldwide industrial demand of quality and efficiency in crop and food production, the importance of phenotyping arises every day. Plant phenotyping refers to a quantitative description of the plant’s physiological, biochemical and morphological properties, among others [1]. It consists of the identification of effects on the phenotype as a result of genotype differences and the environmental conditions to which a plant has been exposed [2]. The development of new usable technologies and its direct availability have driven the latest plant phenotyping approaches that have emerged and have already been applied in several environments [3]. These technologies have enabled the performance of phenotyping tasks with reduced time and monetary costs (much sought after by the industrial actors) and remain under the focus of researchers from different currents of investigation, trying especially to provide realistic, applicable and suitable solutions. Proximal sensing approaches, such as spectroscopy sensors or hyperspectral imaging, have arisen in the last few years as fast, non-destructive resources for the gathering of crop spectral information that could characterize concrete phenotyping traits, providing the in-field methods with a high relevance due to their desirable capability of providing *in situ* results.

Viticulture has benefited from these results of recent research that have developed methods and procedures for several vine- and wine-related problems using near-infrared (NIR) spectroscopy. NIR spectroscopy is a potent technology widely used in several agricultural areas due to its non-destructive nature and multi-parametric capabilities [4]. Spectroscopic sensors have been proven to be fast for the real-time assessment of several grapevine-related traits, such as the grape composition [5], the grapevine petiole nutrient concentration assessment [6] or the identification of grape berry sunburn symptoms [7]. Therefore, the possibility of the use of NIR technology for grapevine phenotyping arises as an attractive and promising tool for precision viticulture, especially when taking into account the fact that this technique is able to characterize more than one parameter using the same spectral measurement.

NIR devices are able to acquire large amounts of spectral data, making it necessary to manage them in efficient and automatic ways. Data mining has become one of the most valuable research fields in the latest few years due to its knowledge discovery power, direct applicability in several areas and, especially, its proven effectiveness in those problems where it is applied. Data mining through, among others, machine learning techniques have provided procedures for both descriptive (characterizations of the properties of the data) and predictive (learning and induction of the data for forecasting) tasks [8,9]. Some of the most widespread applications of predictive techniques are decision trees [10], decision forests [11] and, particularly, artificial neural networks (ANNs) [12] and support vector machines (SVMs) [13], employed in several research areas, such as medicine [14], business and industry [15] or biology [16]. Support vector machines [13,17] are supervised learning methods used for classification and regression through the nonlinear mapping of the input data. SVMs transform the original dataset into a higher dimension using a kernel function and find an optimal separating hyperplane, the best one that maximally separates the samples. Rotation forests [18] are machine learning ensemble methods that make use of several classification trees (hence the name) to build a meta-classifier. A rotation forest can be used both for classification or regression, depending on the kind of tree-based algorithm used. A robust regression tree is the M5 learner [19], which, although not as familiar as other estimation methods in spectroscopy, like partial least squares (PLS) [20], has demonstrated robustness and efficiency in other applications, such as pan evaporation prediction [21], low-flow forecasting modeling [22] or the water level-discharge relationship [23].

Two important grapevine phenotyping topics are varietal discrimination and water status assessment, tasks addressed in the literature and where spectroscopy especially has played a significant part in the last few years. Current varietal discrimination methods have some lack of aspects that are relevant for an industrial point of view, e.g., their need for a highly trained expert or their destructive nature [24]. Water status assessment especially suffers from this last issue, as well as its time and labor-consuming nature, along with the lower representative capacity (limited number of samples measured) derived from it [25]. Grapevine varietal discrimination using spectroscopic data has been recently attempted by hyperspectral imaging under laboratory conditions [24]. Both in-lab or in-field water status assessment via spectroscopy have also been aimed at, attending to several plant water condition indicators, such as stem water potential [26,27], leaf water potential [26,28,29] or leaf stomatal conductance [26]. It is worth highlighting that each and every one of the mentioned studies has one common factor: the use of partial PLS as the model training algorithm. PLS is a widespread statistical technique commonly used in spectroscopy for the regression of chemometric parameters. Qualitative prediction (e.g., discrimination among discrete classes) can also be achieved using PLS (as in [30], where a binary classification is translated into a regression of two natural numbers) or via a purest discrete classification method, like partial least squares discriminant analysis (PLS-DA) [31]. Still, discrimination models built with PLS-based approaches have not yielded remarkable results when taking into account a considerably large amount of classes. Hence, the attractive attempt to apply less often used data mining techniques for the modeling of NIR spectra, thus making it possible to carry fast, in-field solutions for these two grapevine phenotyping approaches into commercial and industrial demands.

The goal of this study was to evaluate the combined use of different data mining techniques along with a non-destructive NIR portable sensor for the in-field grapevine phenotyping of two concrete traits: the variety classification and the estimation of the plant water status.

## 2. Experimental Section

### 2.1. Experimental Layout, Acquisition of NIR Spectra and Reference Measurements

Two experiments regarding grapevine varietal classification and plant water status were conducted.

Both experiments were carried out during late August and early September 2012 in a vineyard located in Vergalijo, Navarra, Spain (latitude 42°27’45.96” N, longitude 1°48’13.42” W, altitude 325 m). Vines of different varieties were planted in 2009 and trained to a vertical shoot-positioned trellis system at 2 × 1 m inter- and intra-row distances, with a north-south row orientation.

For both experiments, spectra acquisition was performed in the field with an integrated portable NIR spectral analyzer (microPHAZIR™, Thermo Fisher Scientific Inc., Waltham, MA, USA) operating in the range of 1600 to 2400 nm with a step of 8.7 nm (a total of 100 data points per spectrum). All spectra were returned by the device in absorbance mode and in this form were used for analysis.

For the grapevine varietal classification, 10 varieties were used: Cabernet Sauvignon, Caladoc, Carmenere, White Grenache, Pedro Ximenez, Pinot Noir, Tempranillo, Treixadura, Viognier and Viura. For each variety, 10 vines and two adult leaves per vine were selected from the mid-upper part of the shoot (Nodes 6 to 12) for the measurement of its spectrum on the adaxial side, making up a total of 20 leaves per variety. Five spectra per leaf (from different positions of the surface) were taken, and their average was considered the final spectrum of that leaf. Therefore, a total of 200 leaves (10 varieties, 20 leaves per variety) were used, and the name of the corresponding variety was linked to each measurement for the training of the varietal classification model.

For the assessment of the grapevine water status, measurements were carried out in six varieties during two days: Godello, Grenache, Pedro Ximenez (29 August 2012; vapor pressure deficit: 0.87 kPa; average temperature: 21.7 °C; average relative humidity: 68%), Carmenere, Marselan and Tempranillo (5 September 2012; vapor pressure deficit: 0.89 kPa; average temperature: 19.8 °C; average relative humidity: 62%). As in the varietal discrimination experiment, 10 vines and two adult leaves per vine were selected from the mid-upper part of the shoot (Nodes 6 to 12) for each variety. A total of 120 leaves (6 varieties, 20 leaves per variety) were measured, thus for the training of the water status assessment model. Spectra acquisition was done on the adaxial side of the leaves. Afterwards, the midday stem water potential *ψ_stem_* (14:00, solar noon) of each leaf was measured as the reference method of water stress [32]. Stem water potential was determined using a Scholander pressure bomb (Model 600, PMS Instruments Co., New York, NY, USA). The selected leaves were driven into dark adaptation by covering them with aluminum foil bags one hour before the *ψ_stem_* measurement.

In order to test the robustness of the algorithms, a second model for water status assessment was developed involving samples of a given variety (to analyze the *ψ_stem_* prediction capability within one variety) acquired at different seasons and vineyards (to test the prediction capability with samples at different phenological stages). Thirty-six leaf spectral measurements of Tempranillo were acquired in 12 August 2015 in a vineyard located in Tudelilla, La Rioja (42°18’17.9208”, −2°7’15.8376”; vapor pressure deficit: 1.74 kPa; average temperature: 26.7 °C; average relative humidity: 53%) using the same procedure as in 2012. A new variety-specific dataset for Tempranillo was built up merging these 36 samples and the 20 samples of Tempranillo taken in 2012, making up a total of 56 samples.

For both variety discrimination and water status assessment, the optical window of the NIR device was fully covered and vinyl gloves were used when taking the measurements in order to avoid contamination from external light and pollutants.

### 2.2. Spectra Pre-Processing and Data Mining Algorithms

#### 2.2.1. Pre-Processing

An outlier detection analysis was performed before any other spectral treatment. It consisted of the following procedure: at measurement time, the acquired spectrum was compared by the sensor with a previously taken grapevine leaf’s spectrum signature and labeled according to whether the signal was from a leaf or not. All spectra that did not belong to a grapevine leaf were treated as outliers and thus removed.

Scatter correction and spectral derivative were applied to the raw spectra, in order to diminish the physical variability between samples because of scatter and to remove both additive and multiplicative effects in the spectra, respectively [33]. Standard normal variate (SNV) followed by de-trending [34,35] was used as a scatter correction method. Afterwards, a Savitzky–Golay smoothing and derivative process [36] was applied with a window size of five and a second-degree derivative.

#### 2.2.2. Data Mining Algorithms

Due to the different nature of the phenotyping features addressed in this work, distinct data mining algorithms for classification and regression were applied for grapevine varietal discrimination and water status assessment, respectively.

##### Varietal Discrimination

For grapevine varietal discrimination, SVMs were used. The *ν*-SVM algorithm [37] was used in this study, implemented in LIBSVM [38], the post-processed spectra data points, linked with its variety label (the class), being the input of the algorithm. A second-degree polynomial kernel and a *ν* value of 0.1 were set as the parameters of the algorithm.

The classifiers evaluation was performed attending not only to the confusion matrix, but also to a deeper analysis involving the true and false positive rates, precision values and receiver operating characteristic (ROC) curves’ area (area under the curve, AUC). The true positive rate refers to the proportion of samples that were discriminated as a specific class among all samples, which truly corresponded to that class (it is similar to the correctly classified percentage divided by 100). The *false positive rate* is the proportion of samples that were classified as a specific class, but belonged to a different one, among all examples that were not of that class. The precision represents the proportion of the samples that were correctly classified in their class among all those that were classified as that given class. The AUC is the measure of the area that lies under the ROC curve. An ROC curve is a graphic representation of true positive *versus* false positive rates by varying a given threshold. ROC curves’ AUC is a common metric for the evaluation of a classifier. A perfect classifier would achieve an AUC of 1, while a system that classifies instances in a random way would obtain a 0.5 value.

##### Water Status Assessment

To address the grapevine water status assessment, regression with rotation forest and M5 trees was applied. The spectral data points were used as the input of the algorithm and each sample’s *ψ_stem_* measurement as the value to predict. *Weka* software, Version 3.6, [39] was used for the development of the regression models.

##### Algorithm Validation

For the assessment of the algorithms’ results, calibration, cross-validation and external testing were carried out. For each one of the experiments, its dataset was divided into two subsets, training and test, comprising 80% and 20% of the original samples, respectively. The test set was never used for the training of any of the models. In the calibration assessment, the models were developed using the training set and validated with the same one. In the cross-validation, a *k*-fold method was performed upon the training dataset with a *k* value of 5 (in order to maintain the 80:20 ratio, obtaining five executions where, in each one of them, the model is trained with 80% of the samples and evaluated testing the remaining 20%). Finally, the prediction results were obtained via an external validation, training and testing the models with the training and test datasets, respectively. The test set was obtained in a stratified way (e.g., the same number of samples for each grapevine variety was selected). For the varietal classification, 160 and 40 samples were assigned to the training and test datasets, respectively. For the water status assessment, the multi-variety models’ datasets contained 96 and 24 instances, respectively, while the Tempranillo-specific models’ datasets involved 45 samples for the training subset and 11 samples for the test subset.

**Figure 1:**
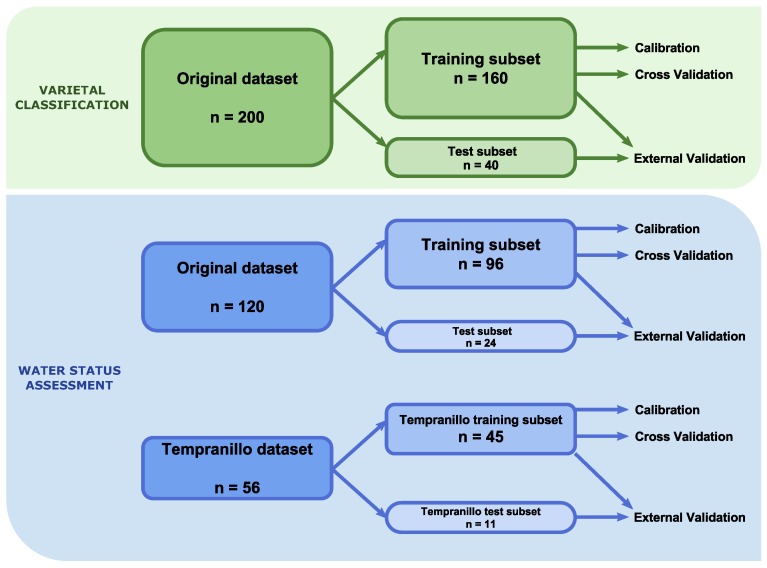
Diagram of the datasets used in both experiments and the different calibration and validation processes.

Figure 1 shows a diagram of the datasets and the different calibration and validation processes used in both experiments.

## 3. Results

### 3.1. Grapevine Varietal Discrimination

The spectral outlier analysis performed before the development of the models resulted in the removal of one sample of White Grenache due to spectral mismeasurement. One hundred percent of correctly classified samples were obtained in the calibration of the SVM classifier trained with the training dataset, reaching perfect scores in the confusion matrix (data not shown). Table 1 presents the confusion matrix from the 5-fold cross-validation of the SVM classifier trained with the training dataset. One hundred forty-one samples out of 159 were successfully discriminated (88.7%). The confusion matrix shows that the Cabernet Sauvignon variety obtained a perfect score in its discrimination, while all other varieties were above the 80% mark, excluding the Viognier variety, which obtained a score of 75% (12 out of 16 correctly discriminated samples).

Table 2 shows a detailed accuracy analysis by class for the SVM classifier in the cross-validation process. Similar to the confusion matrix’s correctly discriminated percentage (Table 1), one class obtained the highest value for the true positive rate (Cabernet Sauvignon). Nevertheless, due to a sample of the Carmenere class incorrectly assigned to the Cabernet Sauvignon class, the precision value of this variety did not reach the maximum. Additionally, although the Treixadura variety did not obtain a perfect true positive score, the fact that no other instance was classified as that variety (Table 1) led to a perfect precision mark for this variety class. The average AUC yielded by the cross-validation was 0.991, moving between 0.976 and 0.999 (achieved by the Treixadura variety) for class-specific values.

Table 3 shows the confusion matrix from the external validation of the SVM classifier, where the model was trained using 159 samples and validated with the prediction of 40 external ones. The average correctly discriminated percentage was slightly higher than that of the 5-fold cross-validation, reaching the 92.5% mark, where 37 out of 40 instances were properly classified. Seven varieties obtained 100% correctly classified samples, while Pinot Noir, Tempranillo and Viognier had one misclassified sample, dropping their percentage to 75%.

The detailed accuracy per class for the external validation is displayed in Table 4. Confirming the results in the confusion matrix of Table 3, seven varieties obtained a value of one in the true positive rate, but two of them—White Grenache and Viura—did not achieve a perfect score, because some samples were misclassified as those varieties. It is remarkable that two classes that obtained a precision value of one (Tempranillo and Viognier) did not reach a full true positive rate, meaning that all samples that were classified as Tempranillo and Viognier were in effect leaves of those varieties. According to the AUC values, eight out of 10 classes yielded the perfect score, increasing the average AUC for all of the classes to the 0.997 mark.

### 3.2. Assessment of Grapevine Water Status

#### 3.2.1. Multi-Variety Model

The spectral outlier analysis tagged one sample of Godello and another of Grenache as mismeasured spectra, so both were removed before the development of the regression model. The ranges of *ψ_stem_* per variety are shown in Table 5. It can be observed that Cermenere and Tempranillo were the most water stressed varieties, while Pedro Ximenez and Godello experienced no water scarcity. Grenache and Marselan exhibited a similar *ψ_stem_* range, indicative of an incipient moderate water stem.

Table 6 shows the statistical summary for the *ψ_stem_* values of the sampled population and the result of the calibration, cross- and external validations of the stem water potential estimation using a rotation forest and M5 trees.

The determination coefficient (R^2^) and root-mean-square error (RMSE) of calibration were 0.97 and 0.083, respectively. For both validation processes, these values were R^2^ = 0.84, RMSE: 0.164, for the cross-validation (having a training dataset with 94 samples) and R^2^ = 0.84, RMSE: 0.165, for external validation (with a test dataset having 24 samples).

**Figure 2:**
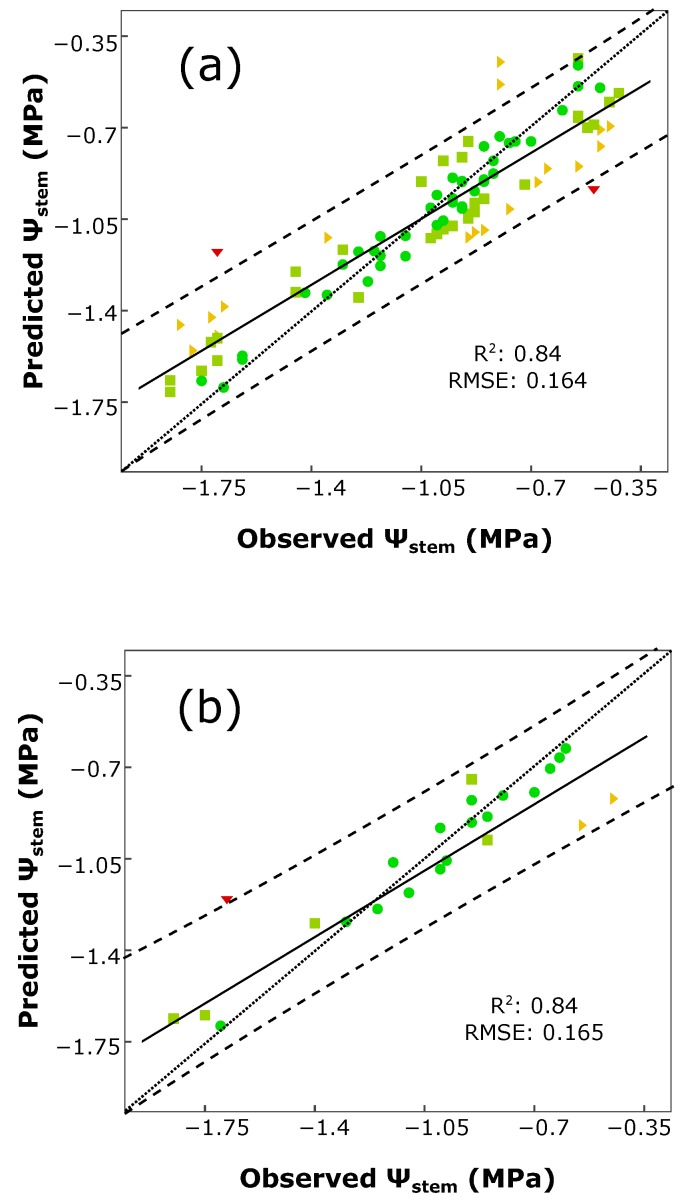
Regression plot for *ψ_stem_* estimation using a Rotation Forest and M5 trees with a 5-fold cross (**a**) and external (**b**) validations. Prediction confidence bands are shown at a 95% level (dashed lines). Solid line represents the regression line and dotted line refers to the 1:1 line. Each points’ color and shape refers to its absolute error value |*ε*| (the absolute value of the difference between the actual value and the predicted one) in MPa: green ●: |*ε*| < 0.1, minimal error; olive ■: 0.1 ≤ |*ε*| < 0.2, low error; orange ►: 0.2 ≤ |*ε*| < 0.4, moderate error; red ▼: |*ε*| ≥ 0.4, high error.

The regression plots for cross- and external validation models are displayed in Figure 2, along with the prediction bands at a 95% of confidence. To perform a deeper analysis of the predicted outcomes, a manual clustering into four groups was performed for the samples according to the absolute error value |*ε*| = measured *ψ_stem_* − predicted *ψ_stem_*: minimal error (|*ε*| < 0.1). low error (0.1 ≤ |*ε*| < 0.2), moderate error (0.2 ≤ |*ε*| < 0.4) and high error (|*ε*| ≥ 0.4). For the cross-validation (Figure 2a), 40 samples obtained a minimal error (42.6% of the samples), 34 a low error (36.2%), 18 a moderate error (19.1%) and 2 a high error (2.1%). In the external validation (Figure 2b), 16 samples obtained a minimal error (66.7%), 5 a low error (20.8%), 2 a moderate error (8.3%) and 1 a high error (4.2%). Two samples for the cross-validation (Figure 2a) and another one for the external validation (Figure 2a) were present in the regression models, samples that obtained an absolute error value greater than 0.4 MPa. In both plots, these high error samples were driven by the divergence of the regression line from the 1:1 line. Further, the samples with the minimal and low error values did considerably fit better to the diagonal line 1:1 than the high error samples. The prediction bands at a 95% confidence only excluded a few samples in both cases. For the cross-validation (Figure 2a), four samples lied out of the precision bands, meaning that 95.7% of the samples were inside both bands. For the external validation results (Figure 2b), 95.8% of the samples lied between the 95% confidence bands, while only one sample was kept out.

#### 3.2.2. Variety-Specific Multi-Vineyard Model

Table 7 shows the statistical summary for the *ψ_stem_* values of the sampled population from the variety-specific model (involving samples of Tempranillo taken both in 2012 and 2015 from two different vineyards) and the result of the calibration, cross- and external validations of the stem water potential estimation using a rotation forest and M5 trees.

The range of *ψ_stem_*, in Table 7, illustrates a population of grapevines involving plants of very different water status, from no stressed plants (*ψ_stem_* = −0.8 MPa) to severely stressed plants (*ψ_stem_* = −1.85 MPa), the mean being *ψ_stem_* (−1.45 MPa), indicative of high water stress.

The determination coefficient R^2^ and RMSE of calibration were 0.92 and 0.098, respectively, while for both validation processes, these values were R^2^ = 0.76, RMSE: 0.159 (cross-validation), and R^2^ = 0.79, RMSE: 0.168 (external validation).

**Figure 3:**
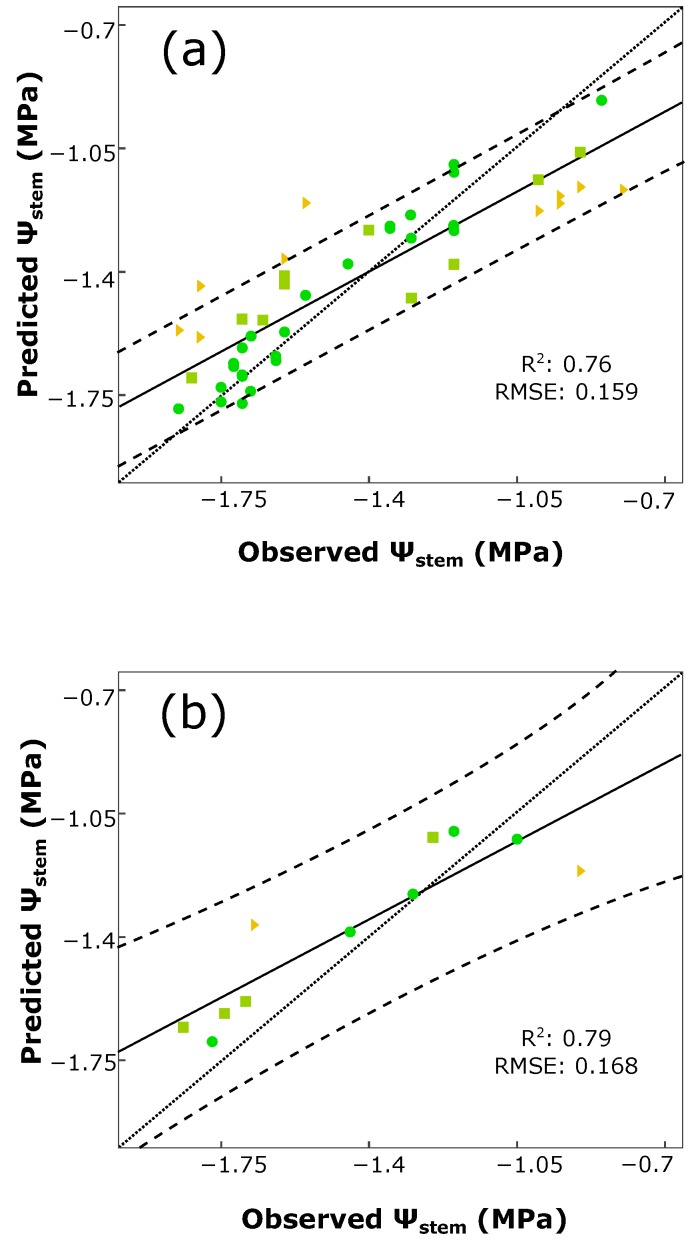
Regression plot for *ψ_stem_* estimation of the variety-specific model (Tempranillo) using a Rotation Forest and M5 trees with a 5-fold cross (**a**) and external (**b**) validations. Prediction confidence bands are shown at a 95% level (dashed lines). Solid line represents the regression line and dotted line refers to the 1:1 line. Each points’ color and shape refers to its absolute error value |*ε*| (the absolute value of the difference between the actual value and the predicted one) in MPa: green ●: |*ε*| < 0.1, minimal error; olive ■: 0.1 ≤ |*ε*| < 0.2, low error; orange ▼: 0.2 ≤ |*ε*| < 0.4, moderate error; red ►: |*ε*| ≥ 0.4, high error.

Figure 3 shows the regression plots for the two validation processes and their prediction bands at a 95% of confidence. In the 5-fold cross-validation (Figure 3a), 25 samples obtained a minimal error value, 10 a low error and 10 a moderate one. Five out of 45 samples lied out the prediction bands, keeping 88.9% of the samples inside them. In Figure 3b (external validation), all of the samples were inside the confidence bands, where 5 of them obtained a minimal error value, 4 a low one and 2 a moderate error value.

## 4. Discussion

In this work, the appraisal of two important phenotyping features in agriculture—grapevine varietal discrimination and water status assessment—has been aimed at from an innovative approach that successfully combines an in-field measurement, using a proximal and non-invasive sensor, with different data mining processing methods. The results obtained have displayed the potential of effectively applying data mining techniques upon the spectral information retrieved from a non-destructive and proximal NIR sensor for grapevine plant phenotyping of two key traits.

Regarding variety classification, most of the widely-used methods for grapevine varietal discrimination have traditionally been either destructive or time-consuming, like classic ampelometry [40] (which is subjected to expert visual description, but still prone to a considerable level of bias due to its human nature), DNA analysis [41] or wet chemistry techniques [42] (carried out by trained people and through destructive methods).

In our work, the 10-class variety classification models using SVMs from non-invasively acquired leaf spectra have yielded 88.7% and 92.5 values of correctly discriminated samples for cross- and external validations, respectively. These high percentages allow one to be reasonably optimistic about the suitability of SVMs for the grapevine varietal classification. These correctly-classified percentages are also supported by the high scores of additional classification statistics, such as the average precision (obtaining in several cases a perfect score and high mean values) and AUCs (an average of 0.991 and 0.997 for cross- and external validation, respectively).

Only very recently, grapevine varietal classification has been attempted by hyperspectral imaging [24] and an NIR portable spectrophotometer [43]. In [24], hyperspectral imaging in the range between 280 nm and 1028 nm was used along with PLS for the classification of 300 leaves from three different varieties (Tempranillo, Grenache and Cabernet Sauvignon), under laboratory conditions. The cross-validation method used (Monte Carlo) yielded more than 92% of correctly classified samples in all cases. The outcomes reached in the present work, even when a large number of varieties was selected for the training, highlights the accuracy shown by data mining techniques for the same goal, particularly when the spectra were collected in the field and in a non-destructive way, different from [24], where a hyperspectral camera was used indoors under controlled illumination conditions. In [43], the authors used a portable NIR spectrophotometer of the same range as the one in this work for the acquisition of leaves’ spectra. Artificial neural networks (ANNs) and sequential minimal optimization for the training of SVMs were tested as classification algorithms for the development of two grapevine discrimination models for two different approaches: a site-specific model for 20 varieties (yielding 87.25% of correctly classified samples, using ANNs) and a global model using six varieties from different vineyards and seasons (obtaining 77.08%, again with ANNs). The higher percentages obtained in the present study could be explained by the selected SVM algorithm, *ν*-SVM algorithm, *versus* sequential minimal optimization, as well as the reduced number of classes.

Varietal discrimination using NIR spectroscopy has also been recently performed for waxy corn seed [44] using SVM and in strawberry [45] and plum [46] using PLS-D. From these works, it is remarkable that a purer data mining technique, SVMs [44], behaved better than the statistical method PLS, commonly used in spectroscopy and chemometrics, confirming the high suitability and adaptability of machine learning approaches for any kind of problem and specifically NIR spectroscopy. Five- and four-class varietal discrimination using PLS-DA was achieved in [45,46] obtaining 69% and up to 96.5% values of correctly classified samples, respectively, presenting lower than and similar accuracies as in the present grapevine varietal discrimination, but taking into account that the number of varieties was reduced by half.

The proven flexibility, generalization capability and accuracy in so many dissimilar fields for discrimination issues given by data mining techniques, and confirmed by the results of the grapevine varietal classification via SVMs, demonstrates how well the numerous data mining algorithms fit in classification problems, specifically when working with NIR spectroscopy from proximal sensors.

Current water status assessment methods are mostly destructive, labor intensive, thus expensive, and, in many cases, only capable of being implemented in a limited number of samples, jeopardizing their representativeness and not suitable for characterizing the spatial variability of a vineyard’s water status. Therefore, new non-invasive and fast approaches are needed.

For the regression of *ψ_stem_* conducted through rotation forests and M5 trees, the calibration R^2^ and RMSE reached the 0.97 and 0.083 values, respectively, while both validation results were virtually identical (R^2^ = 0.84; RMSE = 0.165). A relatively large divergence between calibration and validation results was found, where the latter’s RMSE nearly doubled that of the calibration. Still, this difference of 0.082 MPa, although, as said, being relatively wide, remains small in absolute terms, particularly when compared to the standard deviation of the population *ψ_stem_* values (0.396), that is almost five-times larger. The high score of the determination coefficient of calibration is an aspect that could be generally expected when using data mining and machine learning techniques. Moreover, the training of decision trees is very sensitive to the examples used as input, having a high importance for the algorithm (that tries to extract underlying rules and correlations between the independent and dependent variables), so high results are likely to be obtained when testing with the same set that the algorithm was trained. The use of the calibration results should be carefully treated when applying data mining algorithms, and they should be contrasted with values that came from validation processes. However, the high results obtained for both cross- and external validations concede a considerable level of confidence in the suppression of any overfitting problem.

Additionally, the good outcomes obtained from the variety-specific model (although slightly lower than the multi-varietal one) show the robustness of the application of data mining algorithms for the accurate prediction of *ψ_stem_* of samples from different seasons and locations when properly training the models with both kinds of examples. This could enable affirming that support vector machines are able to assess the grapevine water status within one variety and to discard the variety as a driving factor in good water status prediction. Still, should the variety-specific model have a higher number of samples and/or a wider range in the water status reference parameter (*ψ_stem_*), the model’s performance would have probably yielded higher R^2^ and RMSE values. It should not be omitted that the Tempranillo dataset, compared to the multi-varietal one, contained a lower number of samples (56 *vs.* 120) and a narrower *ψ_stem_* range ([−1.85, −0.8] *vs.* [−1.85, −0.42], MPa).

Stem water potential, as an indicator of plant water stress, has been previously predicted by NIR-based models developed using PLS regression [26,27,28,29], returning determination coefficients between 0.71 and 0.85 (and error values around 0.1 and 0.2 MPa). The in-field multi-varietal study performed in the current work, using rotation forests and M5 trees, returned a similar determination coefficient for cross- and external validations, highlighting that a considerably higher number of varieties was used. The fact that both studies ([26] and this one) clearly resulted in high *ψ_stem_* correlations from two different and scarcely overlapped NIR regions may drive one to conclude the adequate suitability of NIR spectral measurements from non-destructive sensors in water status prediction.

Models for the grapevine *ψ_leaf_* [28] and *ψ_stem_* in olive trees [27] were developed by VIS/NIR spectroscopy. These works have in common the use of PLS as a model training method, returning moderated values of cross-validation correlation (R^2^ from 0.45 to 0.74) that are noticeably surpassed by the results from the rotation forest and M5 trees models described in this work, allowing one to confirm the effective application of data mining techniques to NIR spectral data for the estimation of *ψ_stem_*, hence the assessment of plant water status. Additionally, it must be highlighted that the spectral range used in this study (1600 to 2400 nm) completely covered the absorption band (O–H) corresponding to the water vibrational band (1940 nm) [47], which could be one of the reasons for the high sensitivity in *ψ_stem_* changes; thus, a good predictive model could be obtained from this spectral range.

To the best of our knowledge, scarce studies have made use of data mining algorithms for water status assessment. In [48], the authors built an artificial neural network for the in-lab relative water content (RWC) estimation from grapevine leaf’s hyperspectral imaging working in the range from 900 nm to 1700 nm. The authors asserted that the generated models (with average absolute error below the 3% mark) were shown to be leaf side, varietal and even clone dependent. Although no direct comparison can be made with the present work, because RWC was used as a water status indicator instead of *ψ_stem_*, both results have displayed the accuracy of the combination of NIR spectroscopy along with data mining and machine learning techniques for the reliable assessment of plant, grapevine specifically, water status.

The selection of a proper estimation method for a concrete algorithm and dataset is crucial for the evaluation of the results. In multivariate chemometrics, a classic approach of performance evaluation has been the dataset split into calibration (or training) and test partitions [49]. Although the use of the same dataset for the training and testing is not generally recommended, because the obtained results are overly optimistic [9], its value could be considered as an upper limit to what may be expected in other settings (e.g., cross- and external validation). *k*-fold and leave-one-out cross-validation methods [50] have been broadly extended in data mining and chemometrics. The selection of *k* = 5 for the cross-validation in the present work, maintaining the 80:20 ratio as in the external validation, can lead to a higher consistency and reliability on the obtained results in both experiments.

It is also remarkable the duality brought by the spectral measurements obtained with the same NIR sensor. The capability of effectively addressing these two grapevine phenotyping traits from a single leaf spectral measurement along with its rapid, non-destructive and in-field nature makes the almost direct implementation of a grapevine phenotyping system on an NIR device a reasonable goal supported by the precision obtained in the developed models and the characterization of concrete and sufficient sets of samples for the training.

## 5. Conclusions

Combining non-invasive sensors and data mining algorithms may be a powerful tool that could allow one to perform grapevine phenotyping tasks and forward the results to the user directly in the field. The proven good behavior of different data mining techniques along with the non-invasive, fast and responsive nature of a portable NIR sensor for the in-field grapevine varietal classification and water status assessment opens a way for the direct application of the models in embedded portable systems.

The two phenotyping traits addressed in this study—grapevine discrimination and water status assessment—deserve major attention in modern viticulture, as they are key factors in breeding, grape quality production and sustainability. Their robust prediction from non-invasively acquired data is expected to have a positive impact in precision viticulture and, extensively, several other agricultural areas. These are very likely to benefit from the application of the results obtained by the prediction models due to their almost direct application of the training process via data mining to a portable NIR device.

## Figures and Tables

**Table 1:** Confusion matrix of grapevine varietal classification using support vector machines and a 5-fold cross-validation. The diagonal of the matrix corresponds to the number of samples that were properly classified. The last column displays, for each variety, the correctly classified percentage (n = 159).

		Predicted Variety										
		CS	CL	CR	WG	PX	PN	TE	TR	VO	VU	%
Actual variety	**CS**	**16**	0	0	0	0	0	0	0	0	0	**100.0**
	**CL**	0	**15**	0	0	0	0	0	0	1	0	**93.8**
	**CR**	1	0	**15**	0	0	0	0	0	0	0	**93.8**
	**WG**	0	0	0	**12**	0	0	1	0	2	0	**80.0**
	**PX**	0	1	0	0	**13**	0	0	0	1	1	**81.3**
	**PN**	0	0	0	1	0	**14**	0	0	0	1	**87.5**
	**TE**	0	0	0	0	0	1	**15**	0	0	0	**93.8**
	**TR**	0	0	1	0	0	1	0	**14**	0	0	**87.5**
	**VO**	0	1	0	0	2	0	1	0	**12**	0	**75.0**
	**VU**	0	0	0	0	1	0	0	0	0	**15**	**93.8**

CS: Cabernet Sauvignon; CL: Caladoc; CR: Carmenere; WG: White Grenache; PX: Pedro Ximenez; PN: Pinot Noir; TE: Tempranillo; TR: Treixadura; VO: Viognier; VU: Viura.

**Table 2:** Detailed accuracy by class of the grapevine varietal classification using support vector machines and a 5-fold cross-validation (n = 159).

Class	True Positive Rate	False Positive Rate	Precision	AUC
**Cabernet Sauvignon (CS)**	1.000	0.007	0.941	0.997
**Caladoc (CL)**	0.938	0.014	0.882	0.997
**Carmenere (CR)**	0.938	0.007	0.938	0.998
**White Grenache (WG)**	0.800	0.007	0.923	0.985
**Pedro Ximenez (PX)**	0.813	0.021	0.813	0.980
**Pinot Noir (PN)**	0.875	0.014	0.875	0.976
**Tempranillo (TE)**	0.938	0.014	0.882	0.997
**Treixadura (TR)**	0.875	0.000	1.000	0.999
**Viognier (VO)**	0.750	0.028	0.750	0.992
**Viura (VU)**	0.938	0.014	0.882	0.992
Weighted average	0.887	0.013	0.888	0.991

AUC: area under the receiver operating characteristic (ROC) curve.

**Table 3:** Confusion matrix of grapevine varietal classification using support vector machines and an external validation of 40 samples. The diagonal of the matrix corresponds to the number of samples that were properly classified. The last column displays, for each variety, the correctly classified percentage (n = 40).

		Predicted Variety										
		CS	CL	CR	WG	PX	PN	TE	TR	VO	VU	%
Actual variety	**CS**	**4**	0	0	0	0	0	0	0	0	0	**100.0**
	**CL**	0	**4**	0	0	0	0	0	0	0	0	**100.0**
	**CR**	0	0	**4**	0	0	0	0	0	0	0	**100.0**
	**WG**	0	0	0	**4**	0	0	0	0	0	0	**100.0**
	**PX**	0	0	0	0	**4**	0	0	0	0	0	**100.0**
	**PN**	0	0	0	0	0	**3**	0	0	0	1	**75.0**
	**TE**	0	0	0	0	0	1	**3**	0	0	0	**75.0**
	**TR**	0	0	0	0	0	0	0	**4**	0	0	**100.0**
	**VO**	0	0	0	1	0	0	0	0	**3**	0	**75.0**
	**VU**	0	0	0	0	0	0	0	0	0	**4**	**100.0**

CS: Cabernet Sauvignon; CL: Caladoc; CR: Carmenere; WG: White Grenache; PX: Pedro Ximenez; PN: Pinot Noir; TE: Tempranillo; TR: Treixadura; VO: Viognier; VU: Viura.

**Table 4:** Detailed accuracy by class of the grapevine varietal classification using support vector machines and an external validation of 40 samples (n = 40).

Class	True Positive Rate	False Positive rate	Precision	AUC
**Cabernet Sauvignon (CS)**	1.000	0.000	1.000	1.000
**Caladoc (CL)**	1.000	0.000	1.000	1.000
**Carmenere (CR)**	1.000	0.000	1.000	1.000
**White Grenache (WG)**	1.000	0.028	0.800	0.993
**Pedro Ximenez (PX)**	1.000	0.000	1.000	1.000
**Pinot Noir (PN)**	0.750	0.028	0.750	0.972
**Tempranillo (TE)**	0.750	0.000	1.000	1.000
**Treixadura (TR)**	1.000	0.000	1.000	1.000
**Viognier (VO)**	0.750	0.000	1.000	1.000
**Viura (VU)**	1.000	0.028	0.800	1.000
Weighted average	0.925	0.008	0.935	0.997

AUC: area under the receiver operating characteristic (ROC) curve.

**Table 5:** Stem water potential (*ψ_stem_*) ranges per variety.

*ψ_stem_*	Variety					
	Godello	Pedro Ximenez	Grenache	Carmenere	Tempranillo	Marselan
**Min**	−0.90	−0.65	−1.15	−1.45	−1.85	−1.02
**Max**	−0.62	−0.42	−0.85	−1.10	−1.62	−0.85

**Table 6:** Statistic overview and results of the *ψ_stem_* (MPa) estimation using a rotation forest and M5 trees.

Statistics					Rotation Forest and M5 Trees					
					Calibration (n = 94)		5-Fold Cross-Validation (n = 94)		External Validation (n = 24)	
n	Min	Max	Mean	SD	R^2^	RMSE	R^2^	RMSE	R^2^	RMSE
118	−1.85	−0.42	−1.03	0.396	0.97	0.083	0.84	0.164	0.84	0.165

n: number of samples; Min: minimum; Max: maximum; SD: standard deviation; RMSE: root-mean-square error in MPa.

**Table 7:** Statistic overview and results of the *ψ_stem_* (MPa) estimation for the variety-specific model (Tempranillo) using a rotation forest and M5 trees.

Statistics					Rotation Forest and M5 Trees					
					Calibration (n = 45)		5-Fold Cross-Validation (n = 45)		External Validation (n = 11)	
n	Min	Max	Mean	SD	R^2^	RMSE	R^2^	RMSE	R^2^	RMSE
56	−1.85	−0.8	−1.447	0.314	0.92	0.098	0.76	0.159	0.79	0.168

n: number of samples; Min: minimum; Max: maximum; SD: standard deviation; RMSE: root-mean-square error in MPa.
